# Editorial: Exercise-induced neuroplasticity in neurodegeneration diseases

**DOI:** 10.3389/fnins.2023.1296291

**Published:** 2023-09-29

**Authors:** Laikang Yu, Quincy J. Almeida, Ana Filipa Silva, Lingxiao He

**Affiliations:** ^1^Department of Sports Performance, Beijing Sport University, Beijing, China; ^2^Movement Disorders Research and Rehabilitation Centre, Department of Kinesiology, Wilfrid Laurier University, Waterloo, ON, Canada; ^3^Escola Superior Desporto e Lazer, Instituto Politécnico de Viana do Castelo, Viana do Castelo, Portugal; ^4^The Research Centre in Sports Sciences, Health Sciences and Human Development (CIDESD), Vila Real, Portugal; ^5^Research Center in Sports Performance, Recreation, Innovation and Technology (SPRINT), Melgaço, Portugal; ^6^School of Public Health, Xiamen University, Xiamen, China

**Keywords:** exercise, neuroplasticity, neurodegeneration diseases, Alzheimer's disease, Parkinson's disease

## Introduction

With the increasing life expectancy, the incidence of neurodegenerative diseases, such as Alzheimer's disease (AD) and Parkinson's disease (PD), is rising, creating an urgent need for the development therapeutics. Despite the extensive efforts from researchers throughout the world, effective treatments for combating these diseases are still limited since the majority of these treatments only provide transient benefits.

As a low-cost, low-risk, scalable non-pharmaceutical intervention, the role of exercise in ameliorating cognitive impairment has received increasing attention from the research community, with a growing body of evidence confirming the positive effects of exercise on physical and brain health (Woost et al., 2018; Ferrer-Uris et al., 2022). Previous studies support various mechanisms of exercise-induced promotions in neurogenesis, angiogenesis, cell survival, synaptogenesis, and neuroplasticity that may contribute to enhanced cognitive function (Li et al., 2017; Valkenborghs et al., 2019; Mu et al., 2022). Among these, neuroplasticity is a unique adaptive feature of the nervous system that enables neurons to reorganize their interactions in response to intrinsic or extrinsic stimuli and to form and maintain functional neuronal circuits (Chaudhury et al., 2016), which has been reported to be involved in exercise-related improvements in cognitive function (Svensson et al., 2015).

Therefore, this Research Topic aims to explore the benefits of exercise on cognitive impairment and to explore the mechanisms of those beneficial effects at various levels of the brain and nervous system.

## Summary of selected articles from this Research Topic

Ten manuscripts were received for this Frontiers Research Topic. After rigorous review, 5 articles were finally accepted for publication. The contributing 37 authors were from 3 countries, including China, Brazil, and Denmark. The key contents and findings of each paper are as follows:

The study by Frederiksen et al. examined the effect of moderate intensity aerobic exercise on serum neurofilament light (NfL), a biomarker of neurodegeneration, in patients with mild AD. A total of 136 participants were included in the analysis. NfL were measured using the SIMOA^®^ NF-light^TM^ Advantage Kit before and after 16 weeks of moderate intensity aerobic exercise. Their findings failed to support the beneficial effect of the exercise intervention on a single measure of neurodegeneration in AD. Further studies are needed using other types and durations of exercise and other measures of neurodegeneration.

The mini review by Azevedo et al. summarizes the impact of resistance exercise (RE) on memory and cognitive functions, neurotrophic factors, amyloid-β peptide (Aβ) deposition and plaque formation, and neuroinflammation in humans affected by MCI and AD, and animal models of AD. The findings suggest that RE is a promising strategy for reducing Aβ deposition and plaques, neurofibrillary tangles, and neuroinflammation, as well as for increasing levels of neurotrophic factors and neurogenesis, leading to improvements in memory deficits and cognitive decline. Therefore, RE can be proposed for patients with AD, as an alternative and adjuvant therapy, as a possible therapeutic strategy, not only to improve symptoms, but also to prevent or control the progression of neurodegeneration in AD.

Campos et al. investigated the effects of RE, climbing a ladder with progressive overload in alternate days during 4 weeks, on the molecular (Aβ protein, microglia cells, plasma corticosterone levels) and behavioral (hyperlocomotion, recognition memory impairment) alterations related to AD observed in APPswe/PS1dE9 (APP/PS1) mice. Their results showed that the intermittent program of RE was able to increase central crossings in the open field test and the number of hippocampal microglial cells, and reduce hippocampal Aβ plaques, plasma corticosterone levels, and hyperlocomotion in APP/PS1 mice.

Zhao et al. made a commentary on “*Effects of rhythmic auditory stimulation on motor function and balance ability* in *stroke: a systematic review and meta-analysis* of clinical *randomized controlled studies*” (Wang et al., 2022) published in Frontiers in Neuroscience. They pointed out that two eligible studies that met the inclusion and exclusion criteria were not included and introduced an inverse variance heterogeneity (IV-het) model that could be applied to highly heterogeneous outcomes to validate the true effect size of the outcome. Their results showed statistically significant advantages for the intervention group in terms of step length, step cadence, and velocity, confirming the conclusion of authors.

The study by Yang et al. explored the effects of Qigong exercises on upper extremity muscle activity, balance function, and quality of life in stroke patients. A total of 41 patients with a clinical diagnosis of stroke were recruited. Neurological factors and biofeedback effects, balance, and quality of life were assessed using surface electromyography (sEMG), the PK254P balance function detection system, and the World Health Organization Quality of Life-Brief version (WHOQOL-BREF) scale at pre-intervention, 4 weeks post-intervention, and 8 weeks post-intervention, respectively. Their results showed that 8 weeks of Qigong exercises significantly improved physical health, psychological health, and quality of life in stroke patients and had positive effects on the coordination of limb extremities and balance function.

Collectively, these published manuscripts in the current issue have contributed meaningful novel findings. Yet, considering the limited number of available studies, more research on the correlations between neuroplasticity and exercise characteristics, such as type, dosage, and frequency, are still needed. As editors of this Research Topic, we are very grateful to the authors for their contributions and hard work in scientific research. We hope that the readers will be inspired and utilize the information to advance their scientific pursuits.

## Author contributions

LY: Writing—original draft, Writing—review and editing. QA: Writing—review and editing. AS: Writing—review and editing. LH: Writing—review and editing.

## References

[B1] Chaudhury S. Sharma V. Kumar V. Nag T. C. Wadhwa S. (2016). Activity-dependent synaptic plasticity modulates the critical phase of brain development. Brain Dev. 38, 355–363. 10.1016/j.braindev.2015.10.008 26515724

[B2] Ferrer-Uris B. Ramos M. A. Busquets A. Angulo-Barroso R. (2022). Can exercise shape your brain? A review of aerobic exercise effects on cognitive function and neuro-physiological underpinning mechanisms. AIMS Neurosci. 9, 150–174. 10.3934/Neuroscience.2022009 35860684 PMC9256523

[B3] Li Y. Zhao L. Gu B. Cai J. Lv Y. Yu L.. (2017). Aerobic exercise regulates Rho/cofilin pathways to rescue synaptic loss in aged rats. PLoS ONE. 12, e0171491. 10.1371/journal.pone.0171491 28152068 PMC5289643

[B4] Mu L. Cai J. Gu B. Yu L. Li C. Liu Q. S.. (2022). Treadmill exercise prevents decline in spatial learning and memory in 3 × Tg-AD mice through enhancement of structural synaptic plasticity of the hippocampus and prefrontal cortex. Cells. 11, 244. 10.3390/cells11020244 35053360 PMC8774241

[B5] Svensson M. Lexell J. Deierborg T. (2015). Effects of physical exercise on neuroinflammation, neuroplasticity, neurodegeneration, and behavior: what we can learn from animal models in clinical settings. Neurorehabil. Neural. Repair. 29, 577–589. 10.1177/1545968314562108 25527485

[B6] Valkenborghs S. R. Noetel M. Hillman C. H. Nilsson M. Smith J. J. Ortega F. B.. (2019). The impact of physical activity on brain structure and function in youth: a systematic review. Pediatrics. 144, e20184032. 10.1542/peds.2018-4032 31554668

[B7] Wang L. Peng J. L. Xiang W. Huang Y. J. Chen A. L. (2022). Effects of rhythmic auditory stimulation on motor function and balance ability in stroke: a systematic review and meta-analysis of clinical randomized controlled studies. Front Neurosci. 16, 1043575. 10.3389/fnins.2022.1043575 36466174 PMC9714437

[B8] Woost L. Bazin P. L. Taubert M. Trampel R. Tardif C. L. Garthe A.. (2018). Physical exercise and spatial training: a longitudinal study of effects on cognition, growth factors, and hippocampal plasticity. Sci. Rep. 8, 4239. 10.1038/s41598-018-19993-9 29523857 PMC5844866

